# Clarifying responsibility: professional digital health in the doctor-patient relationship, recommendations for physicians based on a multi-stakeholder dialogue in the Netherlands

**DOI:** 10.1186/s12913-021-07316-0

**Published:** 2022-01-30

**Authors:** Anna V. Silven, Petra G. van Peet, Sarah N. Boers, Monique Tabak, Aviva de Groot, Djoke Hendriks, Hendrikus J. A. van Os, Tobias N. Bonten, Douwe E. Atsma, Tycho J. de Graaf, Mirjam P. Sombroek, Niels H. Chavannes, María Villalobos-Quesada

**Affiliations:** 1 Department of Public Health and Primary Care, Leiden University Medical Centre, Hippocratespad 21, 2333 RC Leiden, the Netherlands; 2 National eHealth Living Lab (NeLL), Leiden, the Netherlands; 3 Department of Medical Humanities, Julius Centre for Health Sciences and Primary Care, University Medical Centre Utrecht, Utrecht, the Netherlands; 4 Department of Biomedical Signals and Systems, Faculty of Electrical Engineering, Mathematics and Computer Science, University of Twente, Enschede, the Netherlands; 5 eHealth Group, Roessingh Research and Development, Enschede, the Netherlands; 6 Tilburg Institute for Law, Technology, and Society, Tilburg University, Tilburg, the Netherlands; 7 Vitrec, BVI Medical, Vierpolders, the Netherlands; 8 Department of Cardiology, Leiden University Medical Centre, Leiden, the Netherlands; 9 Civil Law, Leiden Law School at Leiden University, Leiden, the Netherlands; 10 Health Law, Leiden Law School at Leiden University, Leiden, the Netherlands

**Keywords:** Digital health, Professional digital health, Responsibility, Liability, eHealth, Health policy

## Abstract

**Background:**

Implementation of digital health (eHealth) generally involves adapting pre-established and carefully considered processes or routines, and still raises multiple ethical and legal dilemmas. This study aimed to identify challenges regarding responsibility and liability when prescribing digital health in clinical practice. This was part of an overarching project aiming to explore the most pressing ethical and legal obstacles regarding the implementation and adoption of digital health in the Netherlands, and to propose actionable solutions.

**Methods:**

A series of multidisciplinary focus groups with stakeholders who have relevant digital health expertise were analysed through thematic analysis.

**Results:**

The emerging general theme was ‘uncertainty regarding responsibilities’ when adopting digital health. Key dilemmas take place in clinical settings and within the doctor-patient relationship (‘professional digital health’). This context is particularly challenging because different stakeholders interact. In the absence of appropriate legal frameworks and codes of conduct tailored to digital health, physicians’ responsibility is to be found in their general duty of care. In other words: to do what is best for patients (not causing harm and doing good). Professional organisations could take a leading role to provide more clarity with respect to physicians’ responsibility, by developing guidance describing physicians’ duty of care in the context of digital health, and to address the resulting responsibilities.

**Conclusions:**

Although legal frameworks governing medical practice describe core ethical principles, rights and obligations of physicians, they do not suffice to clarify their responsibilities in the setting of professional digital health. Here we present a series of recommendations to provide more clarity in this respect, offering the opportunity to improve quality of care and patients’ health. The recommendations can be used as a starting point to develop professional guidance and have the potential to be adapted to other healthcare professionals and systems.

**Supplementary Information:**

The online version contains supplementary material available at 10.1186/s12913-021-07316-0.

## Background

Digital health,^1^ also known as eHealth, plays an important role in healthcare systems throughout the world [4–6]. Its potential was clearly proven during the COVID-19 pandemic [7, 8]. Examples include electronic patient records, telehealth programmes, health apps and digital monitoring systems. Beyond current applications, digital health is often presented as a solution to relevant healthcare challenges, such as increasing costs, the ageing population and the growing resource gap [6, 9, 10]. As a result, this sector has been stimulated at national and supranational levels and it has been boosted by strong investments. Despite high expectations, implementation and adoption of digital health is not always successful. This situation has also been reported in the Netherlands, although it is considered by some as a ‘frontrunner’ in digital health [11, 12]. In 2019, for example, it was published that the offer of digital health solutions in the Netherlands was bigger than the application of these tools in daily practice [12]. In order to improve the implementation and adoption of digital health, many efforts have been made to gain insight into barriers and facilitators of digital health, including organisational, scientific and financial aspects [13–15].

At this stage, it has become clear that implementation of digital health technologies (DHTs) in clinical practice goes beyond developing a new technique. The introduction of these types of new technologies generally involves adapting pre-established and carefully considered processes or routines in a complex healthcare landscape [16]. Additionally, innovation cannot be driven by technology alone but needs to be guided by the necessities of end users, and importantly, by our social values. In that respect, analysis of legal and ethical issues regarding digital health are an integral part of the innovation process [17–19]. These issues are related to fundamental rights, such as, but not limited to, privacy, autonomy, justice and non-discrimination concerns. Where legal and ethical dimensions of digital health are under scrutiny there is a growing need for practical and actionable perspectives, which could contribute to closing the gap between theory and practice [20, 21].

The general aim of this study was to identify key ethical and legal dilemmas regarding the implementation of digital health in the Netherlands, and subsequently understand and propose actionable solutions to the most pressing obstacles. This process was approached from a multidisciplinary, empirical and context-dependent perspective. Here, the results of a series of focus groups are presented in which the most pressing and current issues regarding responsibility and liability were identified. Finally, recommendations which could provide more clarity with respect to physicians’ responsibility are offered in order to support the implementation of digital health in clinical practice. These recommendations could potentially serve as the basis to formulate professional guidance for physicians, and could be adapted to fit other healthcare professionals and/or settings.

## Methods

Between March 2018 and November 2019, a series of multi-stakeholder sessions were organised at independent special event locations in Leiden, the Netherlands. Stakeholders from all regions of the Netherlands with relevant expertise in relation to the topic and who were either directly or indirectly working with digital health were invited via email. All stakeholders invited were given the opportunity to propose other experts to be invited. To stimulate patient involvement, a representative of the Netherlands Patient Federation was also invited. Eventually, five out of 56 invited participants were not able to attend due to other obligations or illness.

An opening meeting was organised to identify the general challenges within three predefined subjects: (a) responsibility and liability, (b) good use and (c) governance and privacy. According to their experience or subject of preference, participants were divided into three separate semi-structured brainstorm sessions [22]. In this paper, the findings regarding the first topic will be discussed.

After the opening meeting, two consecutive follow-up focus groups composed by ten to twelve stakeholders were organised to discuss responsibility and liability in the context of digital health. Each session lasted two hours and was supervised by different moderators (PP, AS, ET) and collaborators (NK, MP, AS). The moderators were physicians who had first-hand experience with digital health. The sessions had a semi-structured format, where the moderator facilitated cases in order to promote and guide the discussion, but did not limit the scope of the dialogues (the discussion guide developed for this study is provided as supplementary material I). Each focus group was transcribed during the meeting by an independent assistant (NK, DB, CS). After each session, the participants received a summary and they were able to send any additional comments via email.

The transcripts of the sessions were analysed by two independent researchers (MV and AS) who produced an analytical report. Through a thematic analysis, a central narrative in the discourses was identified [23]. Based on this data, the researchers derived a series of recommendations. Through respondent validation, the results of the thematic analysis and the recommendations were aligned with participants’ views. The feedback received from participants during this process greatly enriched the output of this study. Recommendations were regarded as the most suitable way of communicating the output of this study because the main goal established by the participants was to solve practical issues.

## Results

Participants included ethicists, physicians, lawyers, experts in digital health, and representatives of technology companies, medical associations and regulatory bodies. Two thirds of the participants were women and one third was men.

### Scope and general findings

At the beginning of the focus groups, the necessity of a clear definition of digital health was discussed, resulting in the use of the working definition as presented in footnote 1. Next, participants characterised the subject ‘responsibility and liability’ to be broad and complex (quote 1; all quotes are available in the supplementary material II). Responsibility and liability were found to be closely related concepts that reflect the obligation of a person to behave correctly towards another party, which leads the person to become accountable for that behaviour [24]. During the focus groups, these notions were further clarified and placed in the context of the main themes and subthemes.

### Main theme

The overall emerging theme was: ‘uncertainty regarding responsibilities’. Participants mentioned numerous examples illustrating that stakeholders involved in the implementation and adoption of digital health often feel insecure about their responsibilities (quote 2). This particularly impacts the doctor-patient relationship and may affect the physicians’ role of promoting health and not causing harm. Physicians are also concerned of being held liable for possible harm done to a patient’s health, which could cause reluctance when adoptining digital health in clinical practice (quote 3).

The group identified factors that contribute to the lack of clarity regarding responsibility: having little experience in or knowledge of digital health, lack of accessible information about the particular digital health tool, difficulty to clearly and/or uniformly interpret the available information, little specific professional guidance, and difficulties to access specialised advice, e.g. from a legal advisor (quote 4). Other issues posed included the challenge of physicians to find the appropriate technology among the broad offer, and to subsequently determine its suitability based on an evaluation of risks and benefits according to a patient’s specific circumstances. The group considered that often, physicians encounter limited high-quality evidence for digital health applications. The (peer-reviewed) evidence for efficacy and/or effectiveness can be hard to find, incomplete, or not completely generalisable and/or applicable to the particular case at hand. Other factors that prompt hesitation are uncertainties about security and safety measures, technical reliability (e.g. accuracy and trustworthy generation of data), appropriate incorporation of data generated through patient-initiated digital health into the clinical decision-making process, system’s or patient’s autonomous decision-making, technical knowledge, and (digital) health literacy of both patient and physician (quote 5).

Because of the complexity of settings where digital health can be used, the group narrowed down the scope of the main theme. This was done through setting the scene (subtheme ‘professional digital health’), identifying relevant players (subtheme ‘multiple stakeholders’) and focusing on the perspective of physicians (subtheme ‘responsibility from a physician’s perspective’).

### Professional digital health

It was discussed that responsibility is determined by the settings in which digital health is applied (quote 6). On one end, digital health can be used independently by the patient, for self-care, and without ever interacting with a physician. This is known as ‘consumer digital health’ [25]. On the other hand, digital health can be mediated by care providers within a doctor-patient relationship. This was labelled as ‘professional digital health’ [26]. The participants decided to initially target the latter, since this multidisciplinary, expert driven bottom-up approach was thought to potentially have more impact.

### Multiple stakeholders

**Fig. 1:**
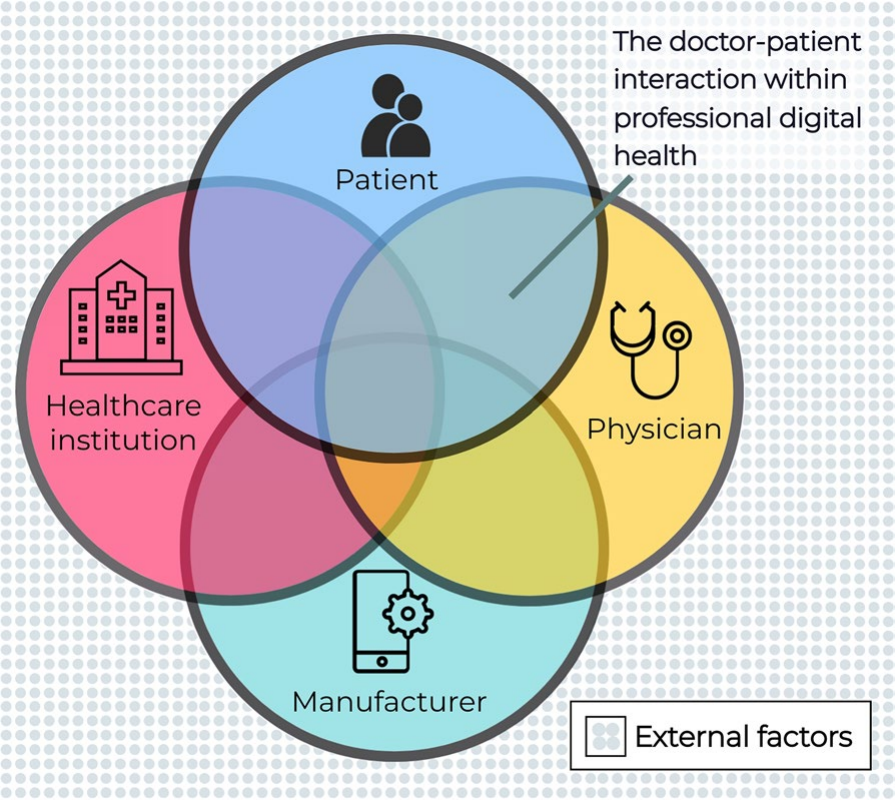
Interacting stakeholders within ‘professional digital health’. Here the roles of patient, physician, the healthcare institution and the manufacturer (including those hosting and processing data) converge. The doctor-patient interaction is key because the decision-making process regarding the adoption of digital health generally occurs in this context. Professional digital health is influenced by the roles of the health institution and the manufacturer, which serve to ensure the quality of the digital health technology and offer technical and organisational support for its application when necessary. In the background, external factors outside of the physician’s control may influence the performance of a digital health technology, such as the quality of the internet connection.

As a next step, professional digital health was analysed. A complicating factor is that professional digital health is a context where several stakeholders with partly overlapping responsibilities interact, making it difficult to delineate who is responsible for what: the patient, the treating physician and other healthcare professionals, the healthcare institution and the manufacturer of the DHT (Fig. 1, quote 7). In the background, external factors outside of the physician’s control may influence the performance of a DHT; e.g. the internet connection and devices that are organised by the patient with private service providers. This complex context may be problematic when attributing liability, e.g. when harm is caused to a patient’s health.^2^ From this point on, we will focus solely on the doctor-patient relationship in order to delineate our scope.

In addition to physicians, patients were recognised to have a prominent role. Digital health, as a viable clinical choice, generally depends on patients performing tasks, making appropriate decisions, and collecting data about their own health, independently or under remote supervision. This way of approaching healthcare supports the ongoing effort to achieve a collaborative management of health. Furthermore, it offers an opportunity to stimulate self-management (especially of chronic diseases) and to inform patients about their own health (e.g. promoting the adoption of healthy behaviours) both of which have been proven to be effective interventions (quote 8) [27].

Another frequent issue identified during the discussions was the worry that, in some cases, patients’ expectations do not match the intended use of a DHT. Participants considered that patients need to receive appropriate information, which will contribute to creating accurate expectations and allowing patients to take responsibility in the setting of professional digital health (quote 9). It was emphasised that patients need to be able to apply the information received in practical situations regarding their own health, without direct guidance. If this is the case, evaluating the suitability of digital health needs to address the patient from a multidimensional perspective that includes clinical, psychological, functional and social aspects, and by paying special attention to the patients’ (digital) health literacy. Thus, prescribing digital health requires an excellent communication process to support patients in the process of understanding, learning and adopting digital health.

### Responsibility from a physician’s perspective

The group focused on the perspective of the practicing physician, arguing that physicians could take a central role in the adoption and promotion of digital health. To play this role, physicians need to overcome the elements that generate hesitation in the adoption of digital health. For example, physicians can be liable for incidents, complications or adverse events in cases of misconduct or negligence, when compared to what is expected from an average experienced and competent colleague; in other words: when failing to conform to the standard of care [28, 29]. On top of that, the group pointed out that the increasing ‘claim culture’ is also causing reluctance, negatively impacting the practice of medicine [29].

It was argued that, above all, physicians’ responsibility is to be found in living up to their general duty of care, in other words, to do what is best for patients.^3^ This is related to the notions of not causing harm and doing good for patients; core principles identified in Health Law as the principles of non-maleficence and beneficence (quote 10) [30]. We are aware that there are other approaches to medical ethics, and responsibility and liability can be given other interpretations; however, they were not the primary focus of the dialogues.

Physicians owe a duty of care to their patients, and in professional digital health, it may be particularly difficult for physicians to be sure to act in line with their responsibilities, inter alia because digital health is a rapidly changing field, and in some cases it alters the roles and responsibilities of both physician and patient (quote 11). In comparison to other fields of healthcare, the scope of the physicians’ responsibility towards their patients within professional digital health is less clear; e.g. as established in existing legal frameworks and codes of conduct. Other aggravating factors are the lack of clear guidance, consensus and structural support for physicians (quote 12). In order to facilitate further guidance for this process, recommendations were formulated.

### Recommendations

This multidisciplinary approach to address ethical and legal challenges of digital health aimed to connect theory and practice, thereby further stimulating trustworthy implementation and adoption of digital health. With the goal in mind to translate these findings into practical tools, a set of recommendations for physicians and professional and/or medical associations is offered in the following section. These recommendations also resonate on core ethical principles, rights and obligations as established in relevant legal frameworks. For example, the principles of non-maleficence and beneficence are addressed in the recommendations related to ‘The expected benefits should be greater than the risks’, and the principle of autonomy is reflected in ‘The right to information and obligation to inform should be specified’. The recommendations make the transition from principle to action, clarifying how to fulfil ethical principles, rights and obligations in the particular setting of professional digital health, and could further contribute to the doctor-patient shared decision-making (Table 1).

**Table 1:** Summary of recommendations aimed at clarifying physicians’ responsibilities within the scenario of professional digital health

**The expected benefits should be greater than the risks:**	
a. Professional/medical associations should play an active role in supporting physicians in the adoption of digital health.	
b. When possible, act and prescribe based on the organisational endorsement of evidence-based digital health.	
c. Actively contribute to the establishment of evidence-based digital health.	
d. Carry out a holistic evaluation of the patient, including (digital) health literacy.	
**The right to information and the obligation to inform should be specified:**	
e. Communicate about the intended use.	
f. Communicate about patients’ responsibilities and explain the risks of adopting digital health.	
g. Communicate about the adequate response to unexpected situations.	
h. Communicate the conditions to access the device/app and give security advice.	
i. Communicate the steps to discontinue the use of digital health and offer alternative treatment options.	

### The expected benefits should be greater than the risks

In general, clinical decisions entail potential risks and benefits for each individual patient. The evaluation leading to the conclusion that benefits are significantly greater than the risks may not always be straightforward, even when prescribing validated or endorsed DHTs. The following recommendations focus on supporting the evaluation process performed by the physician.

- *Professional/medical associations should play an active role in supporting physicians in the adoption of digital health.* Although clinical assessments are an integral part of medical practice, the frequently insufficient evidence and standards, the lack of clear professional guidance, and the absence of unified information about DHTs can be problematic for physicians [18]. The provision of an acceptable level of certainty regarding these aspects may contribute to making better and more informed decisions regarding digital health. Professional/medical associations could play a role in improving this situation by:
  i. offering professional guidance to specify the role of digital health in clinical practice;
  ii. facilitating physicians with an overview of DHTs and their characteristics and intended uses, similar to the function of national pharmaceutical formularies^4^;
  iii. contributing to the establishment of evidence-based digital health. This includes clinical and technical evaluations, usability and cost assessments and studies about the impact on end users and society (e.g. science and technology or social sciences studies) [31].
- *When possible, act and prescribe based on the organisational endorsement of evidence-based digital health, by for example, a healthcare institution or a professional/medical association.* Individual physicians cannot be given the responsibility of evaluating the great number and variety of DHTs based on the available evidence. Therefore, we recommend physicians to rely on guidance and recommendations established by their institution or professional/medical associations regarding DHTs when possible. This allows physicians to act according to existing structures based on (inter)national agreement and expert consensus.
- *Actively contribute to the establishment of evidence-based digital health.* Because of their close relationship with patients and their clinical knowledge, physicians have the ability to become the link between digital health innovations and clinical applications (e.g. identifying if and how a DHT can become part of a care pathway). We recommend physicians begin or continue actively participating in implementation and/or research programmes for digital health. Research is an important pillar of innovation and contributes to ensuring the effectiveness of DHTs and their efficient and evidence-based adoption into clinical practice [8].
- *Carry out a holistic evaluation of the patient, including (digital) health literacy.* The suitability of a digital health solution should consider the clinical history, the psychological, functional, social and environmental dimensions of the patient, and the level of (digital) health literacy. This allows physicians to better understand what can be expected of patients once they are back at home, offering physicians the opportunity to determine the suitability of digital health and to establish an adequate implementation strategy (e.g. one that includes training or extra support). In the case of digital health, it may be useful for physicians to consider a gradual introduction, in order for patients to develop the necessary capacities to fully engage in a digital health care pathway. Additionally, physicians should keep in mind that digital health may provide patients with the opportunity to enhance their capabilities and increase their level of autonomy. This holistic approach is neither new, nor specific to digital health: already in traditional clinical settings, physicians must consider if and to what extent patients are capable of understanding and adapting to their situation and care pathway [32].

### The right to information and the obligation to inform should be specified

Within medical practice and Health Law, the right to information and the obligation to inform are established already [33]. However, more support is needed on how to adequately fulfil this duty as a physician, specifically within professional digital health. The following recommendations address this issue and could significantly offer more certainty to physicians, especially if taken into account for the establishment of professional guidance.

e. *Communicate about the intended use.* In order to align patients’ expectations to the actual intended benefits of a digital health solution, it is relevant for patients to understand its intended use and its general goal. Table 2 summarises different types of DHTs, according to the general goal and the system’s level of autonomy. Autonomy is understood as a feature that allows digital systems to perform tasks independently, without the input or control of human operators; and does not refer to autonomy in the ethically relevant sense [34]. Although several frameworks have been proposed to classify DHTs, to our knowledge none were specifically designed to assist physicians in explaining the system’s level of autonomy in relation to the general goal of the intervention [35–37]. Physicians and/or their team need time to make clear how the measurements and/or information generated by patients will be ultimately communicated back to them. It is particularly important for patients to know how and when information is acquired, when it will be analysed, when and to what extent it will be acted upon, and by whom (human or machine).

**Table 2:** Types of digital health technologies, according to general goal and the system’s autonomy level

General goal	Description	System’s autonomy level
**Real-time responder (with automatic responses and automatic warnings)**	Digital health works as an automatic acute responder to the worsening of symptoms or emergency situations that tracks patients 24/7 (e.g. an implantable cardioverter-defibrillator (ICD), which automatically acts upon arrhythmias and additionally transfers real-time data to the healthcare professional).	Very high
**Scheduled monitoring system with automatic warnings**	The digital health technology acquires data in a scheduled manner and issues automatic warnings for the patient and/or health professional when necessary (e.g. a smart medicine box that indicates when to take the medication and alerts patient and/or physician when medication is not taken).	High
**Digital assistant for an independent user**	Digital health guides patients to take action independently, while the physician controls patients’ health through scheduled appointments (e.g. an insulin pump that is fully controlled by the patient, while the physician follows up the patient’s health through planned appointments).	Medium
**Digital registry**	Digital health works as a digital registry that is regularly evaluated by the physician in a scheduled manner (e.g. patients with cardiovascular diseases monitor their physiological parameters from home with a device coupled to their electronic medical records; while their health is reviewed together with their physicians, during scheduled medical appointments).	Medium
**‘Nudger’**	Digital health encourages patients to change behaviour patterns to live healthier, or to prevent or improve a condition (e.g. coaching apps that help with diet, sport, human contact, etc.).	Medium - Low
**Communication tool**	Digital health is used as a communication support tool, in addition to the traditional ways to access medical care (e.g. primary care eConsults).	Low
f. *Communicate about patients’ responsibilities and explain the risks of adopting digital health*. One of the strongest arguments in favour of digital health is the empowerment of patients regarding their own health [38]. In some cases, digital health care pathways require patients to actively perform tasks and take decisions, assigning them a higher level of responsibility. After considering if a patient is capable of engaging in a digital health care pathway and acquiring a new level of responsibility, the physician needs to discuss the responsibilities to be undertaken and the risks entailed. Patients should for example be aware that the information they generate at home will be the basis of their physicians’ clinical decisions. Therefore, if data is generated incorrectly, misreported or not generated at all, the patient may be at risk. The types and degrees of risk that digital health entail are specific to each digital health intervention and each patient, depending on the patient’s characteristics (recommendation ‘d’). In this respect, the role of physicians to carry out a holistic evaluation of the patient continues to be vital.
g. *Communicate about the adequate response to unexpected situations.* When engaging with their patients, physicians should keep in mind that the performance of a DHT can be negatively affected by technical or human factors. Technical factors can be endogenous (e.g. malfunctioning of the app or device) or external (e.g. problems with the internet connection, electricity, the mobile phone or charger). Human errors can be made by any person involved in the digital health care pathway, and they may be challenging to minimise when several people participate (e.g. the patient, caregiver, treating physician, other healthcare professionals, technicians and support personnel). In order to ensure patients’ safety, it is essential for patients (and their caregivers) to be aware of such threats, know (insofar possible) to prevent such threats from arising and, if they do arise, how to respond and how to report them. This can be addressed, for example, through the availability of helpdesks and emergency numbers for patients in case of unforeseen difficulties (additional to the national emergency number).
h. *Communicate about the conditions to access the device/app and give security advice.* The integrity of the app or device should be protected adequately. Two important factors need to be discussed with patients in this regard. Firstly, the device or app should be used only by authorised individuals. Patients need to keep their devices safe; e.g. through the use of passwords or biometric authentication such as fingerprints. In practice however, patients sometimes wish or need to rely on family members or (informal) caregivers when managing their health. Physicians should help patients to find a balance between appropriate security practices and shared health management at home. Secondly, patients should be aware of the level of access they have been granted to apps or devices and the corresponding responsibilities. If patients are granted full access (e.g. to fully personalise the settings of an insulin pump) or if access cannot be restricted by the app or device, physicians should make sure that patients understand the consequences of changing the parameters or settings on their own.
i. *Communicate about the steps to discontinue the use of digital health and offer alternative treatment options.* As with all healthcare options, it is the patient’s right to finally decide if he or she wants to engage in a digital health care pathway, or not. For arriving at this decision, patient and physician should work together and engage in a collaborative effort to consider the advantages and disadvantages (shared decision-making). Additionally, patients should be able to consider all available options (traditional and digital), and understand the opt-out process. There are many reasons why a patient may reject or want to discontinue the use of digital health; e.g. lack of privacy at home, insecurity about the quality of care, the preference for a physical doctor-patient interaction, doubts about their own performance carrying out tasks, etc. [39]. In the perfect scenario, patient and physician should be able to discuss concerns and problems and try to find solutions. However, a patient’s decision to withdraw should be ultimately respected.

## Discussion

### Principal findings

This paper presents the findings of an empirical and multidisciplinary exploration of ethical and legal issues that might occur when engaging in digital health, putting emphasis on physicians’ responsibility when adopting digital health in clinical practice. Responsibility, in this sense, goes beyond the fear of being considered liable. Instead, it is rooted in the duty of care that embraces the core ethical principles of not causing harm and doing good for the patient by ensuring that health is being promoted. Furthermore, it is consistent with the idea of collaborative management of health and the process of shared decision-making that takes place within the doctor-patient relationship. Recommendations were formulated that could contribute to clarify physicians’ responsibility in the setting of professional digital health. It does not intend to strip the responsibility of other players; therefore, we have referred to the responsibilities and roles i.e. of manufacturers, healthcare institutions and patients. Given the increasing importance of providing remote healthcare (e.g. as seen during the COVID-19 pandemic) our recommendations have a great potential to improve the quality of care provided with the help of digital tools.

### Strengths and limitations

To our best knowledge, this is the first study to address the complex and relatively new issue of physicians’ responsibility when prescribing digital health in clinical practice, and to propose actionable solutions. The intensive inter-disciplinary collaboration with experts from different institutes throughout the country allowed for in-depth analyses of dilemmas, leading to realistic and generalisable recommendations. The research was centred in the context of the Netherlands. Nevertheless, it is expected that the findings have parallels with other settings, especially with similar socio-economic contexts. Due to the nature of the study, the number of participants was limited. However, participants have broad practical experience with digital health and we expect to have captured a realistic picture of the national state-of-affairs. The recruitment strategy selected by default those individuals highly interested in digital health, but some of them more critical than others. The focus groups were not recorded, but directly transcribed; to ensure the validity of the analysis and recommendations presented here, emphasis was put on the process of participant validation. Finally, the main discussion focused on physicians’ perspectives only, but these findings can be valuable for other healthcare providers.

### Comparison with literature and other studies

New DHTs are transforming medical practice, but it is challenging for physicians to adapt to these permanently and rapidly changing technological advancements. Applications that impact health and healthcare should be effective and safe, and they deserve, amongst other things, in-depth ethical and legal explorations. A common issue is that such assessments take time but innovation in this field occurs at a very rapid pace. Although legal frameworks governing medical practice already describe core ethical principles, rights and obligations of physicians,^5^ they do not suffice to clarify their responsibilities in the particular setting of professional digital health. There are relevant ongoing efforts that aim to close the legal gaps, e.g. through quality standardisation processes or self-regulation efforts such as codes of conduct [35, 40–43]. In the specific case of responsibility and liability in the context of digital health, emerging dilemmas have been previously identified; however, limited actionable solutions exist [17, 18].

### Implications and ways forward

A key step to ensure the impact of these recommendations (and therefore improve the trustworthy uptake of digital health) is their incorporation in professional guidance for physicians. It is relevant to note that the introduction of DHTs might very well involve the entire healthcare system and other healthcare providers, such as mental health professionals, nurses and caregivers. Consequently, and after a careful analysis of the particular context, these recommendations could be adapted to other professionals, settings and healthcare systems. Further research is necessary to also address this issue from other perspectives (e.g. those of patients) and to carry out an in-depth analysis of the legal framework(s) to identify which gaps could be improved in order to offer physicians legal security.

## Conclusions

The application of digital health in clinical practice shows great potential to improve the quality of healthcare, but its use raises ethical and legal dilemmas that hamper its implementation and adoption. The establishment of practical and generalisable instruments to avoid or overcome these challenges could stimulate a better and trustworthy adoption of digital health, ultimately benefitting patients. As part of a larger study, ethical and legal issues hindering the uptake of digital health in clinical practice were explored empirically with a multidisciplinary group of experts. Here, we have taken a step towards actionable solutions to clarify physicians’ responsibility in the context of professional digital health, while taking into account the roles of the patient, healthcare institutions, medical associations and manufacturers.

## Supplementary Information

**Additional file 1: Supplementary material I.** Routing for the focus groups.

**Additional file 2: Supplementary material II.** Quotations supporting findings, as referenced within the text.

## Data Availability

The transcripts of the focus groups can be made available from the corresponding author upon reasonable request.

## Abbreviations

DHT: Digital health technology
ICD: Implantable cardioverter-defibrillator
GDPR: General Data Protection Regulation
EU: European Union
MDR: Medical Device Regulation
EC: European Commission

## Footnotes

1 **Working definition of digital health. **Digital health (eHealth) is a broad and shifting concept that changes hand in hand with the technological advances. A definition often used is Eysenbach’s: ‘health services and information delivered or enhanced through the Internet and related technologies’ [1]. In order to capture the heterogeneity of digital health, Shaw and colleagues proposed that digital health takes place in three domains: consumer-driven and consumer-controlled technologies (e.g. wearables and apps), digital tools for health stakeholders to interact with each other (e.g. telemedicine and messaging systems), and technologies that improve health and health services through data (e.g. data management systems and repositories) [2]. For our purposes, digital health can be understood as the use of digital technologies to assist or improve health and healthcare [3].

2 In professional digital health, harm can be caused to the patient when, for example:

- a. a device or app malfunctions or does not perform according to the intended use, the technical support of the manufacturer is not available as required or is inefficient, or the manufacturer does not adhere to the contract’s conditions (role of the manufacturer);
- b. the physician makes a clinical decision based on inaccurate data, incorrectly assesses the patient’s competences, or does not follow up the patient appropriately (role of the physician);
- c. the patient does not use the device and/or app appropriately or for the intended use, does not report data as agreed, does not ask for support when necessary, or is not capable of performing the tasks required but does not communicate it to a health professional (role of the patient);
- d. the healthcare institution does not allocate enough personnel or resources to support patients or physicians when using digital health tools, or fails to ensure the quality and optimal performance of devices and/or apps (role of the healthcare institution).

3 Duties of physicians to patients (excerpt), World Medical Association (2006):

- – ‘A physician shall act in the patient’s best interest when providing medical care’.
- – ‘A physician shall owe his/her patients complete loyalty and all the scientific resources available to him/her. Whenever an examination or treatment is beyond the physician’s capacity, he/she should consult with or refer to another physician who has the necessary ability’.

4 See for example the national and publicly available pharmaceutical formulary of the Netherlands: Farmacotherapeutisch kompas. It is an initiative of the National Health Care Institute, accessible online (https://www.farmacotherapeutischkompas.nl/) and via a mobile application.

5 **Legal context regarding health and digital health in the Netherlands**. The Medical Treatment Agreement Act (Wet op de Geneeskundige Behandelingsovereenkomst, WGBO) of the Civil Code regulates the doctor-patient relationship and recognises patient’s rights. Additionally, the Healthcare Quality, Complaints and Disputes Act (Wet kwaliteit, klachten en geschillen zorg, Wkkgz) lays down the quality conditions necessary regarding healthcare and how to proceed when having complaints. The management of sensitive personal data is additionally regulated by the European General Data Protection Regulation (GDPR) and the corresponding Dutch GDPR Execution Act (Uitvoeringswet Algemene Verordening Gegevensbescherming, UAVG). In the European Union (EU), for example, the new Medical Device Regulation (MDR) that is effective since May 2021, came to repeal regulations whose origins are more than 25 years old and pertains to the placing on the market and putting into service of medical devices. It does not however, (directly) specify the responsibilities of the physician vis-à-vis his/her patient when determining the suitability of the medical device, or when prescribing and using such medical devices. In the EU, a multipurpose information technology system called EUDAMED was developed by the European Commission (EC) as part of the implementation of the MDR. The system was recently launched and serves as an interoperable and open system for registration, notification and dissemination to ‘improve transparency and coordination of information regarding medical devices available on the EU market’ (https://ec.europa.eu/health/md_eudamed/overview_en). The practical impact of the MDR and EUDAMED is yet to be seen.
