# Supplementary material for: Clarifying responsibility: professional digital health in the doctor-patient relationship, recommendations for physicians based on a multi-stakeholder dialogue in the Netherlands
Source: BMC Health Serv Res. 2022 Jan 30;22:129. doi: 10.1186/s12913-021-07316-0 (PMC8801038; doi:10.1186/s12913-021-07316-0)
Supplement: Supplementary file 1 — Additional file 1: Supplementary material I. Routing for the focus groups. [file 12913_2021_7316_MOESM1_ESM.docx]

**SUPPLEMENTARY MATERIAL I. Routing focus groups on responsibility and liability concerning digital health**

Focus group 1

*Main question:*

- Which challenges regarding responsibility and liability are encountered when implementing and adopting digital health in clinical practice?

*Other discussion enablers:*

- How is the distribution of responsibility and liability between different (human) parties in Dutch healthcare, specifically for the case of applying digital health in clinical practice?
- How is the distribution of responsibility and liability between human parties and technology in Dutch healthcare?

*The following case was provided to the participants beforehand to illustrate possible dilemmas.*

*Home monitoring* (case #1)
After heart surgery, a patient receives a device with which a single lead electrocardiogram (ECG) can be made. Her healthcare provider (HCP) instructed her to make an ECG at home, on the day before the scheduled video consultation. The HCP then analyses the ECG just before the (weekly) video consultation with the patient. The consultations have been scheduled on beforehand. In addition, the patient was instructed to always call the hospital in case of questions, symptoms or sudden complaints. A week after surgery, the patient suddenly suffers from chest pain. She decides to make an (extra) ECG at home, and fills in the online dairy that she is suffering from some chest pain, but she does not call the hospital. At 4pm the next day, the HCP is preparing the weekly video consultation. However, the HCP then discovers that the patient was admitted to the hospital this morning due to severe heart failure as a consequence of a rhythm disorder. The HCP discovers that the rhythm disorder was already present on the ECG that was submitted the day before.
Possible dilemmas include: are the hospital and/or the HCP responsible for the fact that the patient was admitted to the hospital (too) late? Which role did the use of digital health play here, and what about the role of the patient? What if the device transferred information erroneously to the hospital?

Focus group 2

*Main question:*

- Which challenges regarding responsibility and liability are encountered when implementing and adopting digital health in clinical practice?

*The following cases were provided to the participants beforehand and served as discussion enablers.*

*Malfunctioning alarm (case #2)*Imagine: a patient makes an ECG every week at home. While it should have happened, no alarm signal pops up after the patient measured an irregular rhythm. The HCP analyses the ECG two days before the scheduled appointment. However, the HCP then discovers that the patient was admitted to the hospital due to a rhythm disorder. Now, who is responsible for the possible preventable hospital admission and the malfunctioning alarm?

*If there is extra time, the following question can also be discussed:*

- When using software based on machine learning techniques, it is not always possible to establish how a decision was made. This is also called ‘black box’. Currently, this is leading to a discussion whether or not to use black box algorithms in healthcare. However, scientists have not unravelled yet the exact mechanisms of some often used medications or treatments. How could all stakeholders involved cope with this responsibly?

*Optional discussion enablers:*

*Black box (case #3)*In case of a ‘black box’, an algorithm cannot be used in routine clinical care (algorithms solely being used for the sake of scientific research are outside of scope). To which extent do you agree with this?

*Interpretability of algorithms (case #4)*

HCP should exactly know how an algorithm was built, and how the computer subsequently generated the advice (whether or not with black box). Before an algorithm is going to be used in clinical practice, HCP need to be (demonstrably) educated. To which extent do you agree with this?

Focus group 3

*Main question*:

- Often, the distribution of responsibility and liability between different stakeholders (patient/consumer, healthcare provider/institution, manufacturer, government, etc.) when actively using digital health in clinical practice is not clear. How could the distribution look, and how could we achieve this?
- Which regulation(s) discussing liability in healthcare momentarily exist, and what is not clear yet?

*The following cases were provided to the participants beforehand and served as discussion enablers.*

*‘Conflict’ of authority (#5)*Patient X, 58 years old, bought himself a smartwatch about 1,5 year ago. The smartwatch is, amongst other things, able to measure blood pressure. Patient X travels a lot for his job, mainly sits down during work and sports at an irregular basis. Sometimes he tries to start up jogging again, but it remains difficult to keep up the good habit. He drinks about 15 units of alcohol each week, particularly during diners with clients. He does not smoke. Since a few months, he measures his blood pressure daily. He asks himself whether his blood pressure is too high – his systolic blood pressure ranges between 135 and 185 mmHg, diastolic between 85 and 100 mmHg. The mean blood pressure over the past months is 150/89 mmHg, according to the smartwatch. He decides to visit the general practitioner (GP). The GP is in doubt: should she start treatment according to the mean values of the watch? Is she supposed to ‘trust’ the watch? What should she actually know about these kind of devices, is there some place where she can look up facts like validity and accuracy?

*Disciplinary law (#6)*Patient Y, an elderly women living alone, suffers from heart failure. Her cardiologists indicates that he no longer thinks it safe for her to live alone. However, the patient insists on staying at home. She arranges an digital health service called ‘Health Buddy’ herself. It is a device with a screen and four buttons, which asks the patient questions about her symptoms at a regular basis. The answers are transferred to the cardiologist. The cardiologist is not very enthusiastic about this idea, and tells the patient that he cannot be held responsible for possible failure of the Buddy service. In addition, he insists on having a video consultation once per week. At some time, the patient gets ill. She is unable to answer Buddy’s questions, leading to the cardiologist not being informed about her complaints. That same day the patient dies due to heart failure. Afterwards, the family discovers that she tried to contact the cardiologist via the video consultation service. The family of the patient starts a civil lawsuit and charges him via disciplinary law. Is the cardiologist in some way responsible for the death of the patient?

*Malfunctioning of device (#7)*Patient Z, suffering from lung fibrosis, is recommended to keep track of his oxygen saturation levels at home. Patient Z uses oxygen supply now and then, but he finds it hard to determine the right amount of flow. The pulse oximeter recommended by the doctor should be charged regularly. After a few weeks of use, the charger suddenly catches fire. A small flame arises in the patient’s home. The manufacturer of the device did not supply a warning for this in the instructions for use. The device was allowed on the market properly, possessing CE-certification for medical devices. The patient’s insurance company files an insurance claim to the hospital on the patient’s behalf. Who was actually responsible in this case? What are the duties of the doctor and the hospital in such cases?
