# Supplementary material for: Clarifying responsibility: professional digital health in the doctor-patient relationship, recommendations for physicians based on a multi-stakeholder dialogue in the Netherlands
Source: BMC Health Serv Res. 2022 Jan 30;22:129. doi: 10.1186/s12913-021-07316-0 (PMC8801038; doi:10.1186/s12913-021-07316-0)
Supplement: Supplementary file 2 — Additional file 2: Supplementary material II. Quotations supporting findings, as referenced within the text. [file 12913_2021_7316_MOESM2_ESM.docx]

**SUPPLEMENTARY MATERIAL II. Quotations and notes from focus groups**

Focus groups were carried out in Dutch. The following information has therefore been translated to English by the authors. Focus groups were transcribed during the meetings. For this reason, some quotes are offered in third person, or as a group view that resulted from a joint active discussion between participants. The background of participants is indicated between brackets to note the value of the different perspectives.

1. Background. Participants were posed with the question of how to assign or share responsibility between different stakeholders involved in digital health. This was further developed hand in hand with use cases (supplementary material I). A discussion arises about the difference between (moral) responsibility and (legal) liability:
   Group: ‘*Participants propose to include both responsibility and liability to answer this question [referring to the aforementioned use case]. It is not very clear what the difference between those concepts is, exactly. In other countries, there is also a difference made [between these concepts]. In the Netherlands responsibility is more normative.’*

   Participant (technology): ‘*A difference needs to be made between responsibility and liability. Liability is something that can, in the end, only be determined by a judge; a doctor cannot exempt accountability by him/herself.*’
2. Participant (law): ‘*We’ve never actually established with everybody how responsibility works when it concerns the application of digital health.’*

Participant (health care): *‘Until now the responsibility has been given to the doctor. Digital health is to give more responsibility to the patient’.*

Participant (law): *‘The question is who closes a contract with whom and about what? In a doctor-patient relationship, responsibility cannot be shaken off.’*

1. Background. Fragment of a discussion between two participants about responsibility of physicians and whether they should continuously look at data generated by patients who monitor at home, or whether they can only have a look at certain times.
   Participant (technology): *‘Would you like to be responsible [as a physician, for everything a patient measures at home]?’*.
   Participant (health care): *‘The question is generated from fear, at the end is the doctor the one that stands in front of the judge.’*

Group: *‘... could the physician be held liable by the patient or family [if something goes wrong when engaged in a digital health care pathway]? In theory, that is possible, and can hinder the good implementation of digital health. This is a risk that at this moment needs to be considered regarding [the implementation of] new applications, where it has to constantly be checked with patients how is it going [regarding the use of digital health applications].’*

1. Participant (law) *‘Do you expect the doctor to know enough about the ICT? I don’t think that is realistic…’*
   Group: Several participants agreed on the point that: ‘*it happens often that physicians do not have knowledge about new devices’.*

   Background. Following a discussion on how doctors can determine the accuracy of devices.
   Group: *‘It is impossible to express which blood pressure monitors are good within the law, because it is dependent on the way it is being used. An organisation like the Heart Foundation could do so, for instance. But, how will we determine whether a device is trustworthy? Could we state that devices with a higher risk classification [so that they are subject to the MDR] are generally trustworthy, because they have been tested in scientific research? Can we trust the MDR enough, and the interpretation of [scientific research] results by the notified bodies?’*
2. Participant (technology): *‘Data flows are also getting more complex, the physician gets feedback regarding an [clinical] outcome while the black box did something there. [...] How can the physician know that what the algorithm does? If we are talking about self-learning systems who is responsible?’*

Group: ‘*Devices that meet the requirements and will be put on the market in the following years, will lead to people measuring [their health] more and more. Those measurements will be brought to a doctor. The doctor could say: this wasn’t measured with my validated blood pressure monitor, so I don’t trust it. However, it seems better to recognise the value of these types of trend measurements by saying: we have a history of measurements, showing a trend, this might be more valuable than all measurements that have been done at the practice, with the blood pressure monitor of the practice.* It seems there is a conflict of authority. *It is important to differentiate between people that start using a device on their own initiative and people who do this because a doctor advised them to do so*.’

1. Group: ‘*With the establishment of the quality / trustworthiness of the DHT, we’re not there yet, because there is a complete working environment around it. You can qualify an app as trustworthy, but it should be worked with properly as well.’*
2. Background. The group was presented with case #2 (supplementary material I): A patient makes an electrocardiogram (ECG) weekly according to the plan made with his/her physician. If there is an abnormal heart rate, the system will generate an alarm to warn the patient, who can contact a healthcare professional. In this case, before one of the patient’s appointments, the nurse in the hospital checks the patient’s data and realises that the patient has been admitted to the hospital with a severe cardiac arrhythmia. The system failed to generate the warning alarm that could have avoided the patient developing the severe arrhythmia and being admitted to the hospital. Who is responsible for this?
   Group: *‘This depends on many factors. Why did the alarm fail to sound? Were the settings wrong? If that is the case, whose fault was that? Did that happen because the patient played with the settings? Should it always be possible to know when a patient changed the settings? Did the Internet fail? If the Internet connection has such a big impact, is it not necessary for the device to work offline? There is also a chance that something went wrong from the side of the manufacturer, and this may influence this case. It is important to define what is the use that according to the manufacturer can be given to the device (intended use). It may be the case that the app can only be use in non-urgent situations. The manufacturer is also responsible for instructing users appropriately. If the app is used in a situation that is not meant to, then the responsibility is not the manufacturer’s, and the question will be: who told the patient that he/she may/could use the app (the most logical answer is: the physician).’*
3. Participant (health care): *‘The younger generation has a different idea of responsibility, they want to do and decide more by themselves’.*

Background. Fragment of a discussion between two participants:
Participant (law): *‘There is no universal solution, there must be someone who can tell the patient what is possible and is not. That is the role of the physician’*.
Participant (end-user): *‘Actually [this is decided by] the patient together with the physician or his/her environment.’*Group: *‘[This is] good shared decision making’*

1. Participant (end-user): ‘*You need to manage expectations beforehand.’*

   Participant (health care): ‘*The use of digital health means that patients need to take more responsibility.* *’*

   Participant (law): ‘*With the introduction of technology, expectations are created… you have to communicate about it and make agreements.’*
2. Participant (law): ‘*If they* [physicians] *can motivate their choices and treatment proposal, they live up to the most important principles of Health Law and their duty of care.’*
3. Group: ‘*Rapidly, many new different tools are being developed, with which patients can measure all sorts of things from home; this offers chances, not only disadvantages. However, the developments occur more rapidly than general practitioners and in-hospital physicians can keep up with*.’
4. Group: ‘*It would be good if all DHTs that have been checked/verified could be saved in a database so that a doctor can check whether he/she can trust the measurements/trends observed.’*

Group: *‘...the medical sector needs to be clear and say: there are a lot of apps that we do not want to endorse (non-medical apps)’.*

Participant (law): *‘There are very few certified apps. 270 medical apps from which 27 have been researched approved’.*
